# Intersection of substance use and cardiac arrest mortality in the United States: a 25-year CDC WONDER analysis

**DOI:** 10.1097/MS9.0000000000005685

**Published:** 2026-10-07

**Authors:** Taha Alam, Sohaima Kamal, Syed Hasham Ali, Fatima Ibrahim, Ramesha Tahir, Noor Un Nisa, Neeraj Kumar, Iman Osman Abufatima, Norina Usman, Muhammad Shaheer Bin Faheem, Waqas Burney, Noorulain Aqeel, Shaheer Bin Shafiq

**Affiliations:** a Department of Medicine, Dow University of Health Sciences, Karachi, Pakistan; b Department of Medicine, Bahria University, College of Medicine, Islamabad, Pakistan; c Department of Medicine, Jinnah Sindh Medical University, Karachi, Pakistan; d Department of Medicine, University of Medical Sciences and Technology, Khartoum, Sudan; e Department of Internal Medicine, Adventist Hospital Lodi, Lodi, CA, USA; f Department of Medicine, Karachi Institute of Medical Sciences, KIMS, Karachi, Pakistan; g Department of Family Medicine, Dignity Health Methodist Hospital, Sacramento, CA, USA; h Department of Psychiatry, Napa State Hospital, Napa, CA, USA; i Department of Research, California Institute of Research and Technology, Davis, CA, USA; j Department of Psychiatry, California North State University, Elk Grove, CA, USA; k Department of Psychiatry, Touro University, Vallejo, CA, USA

**Keywords:** cardiac arrest, CDC WONDER, disparities, substance use

## Abstract

**Background:**

Cardiac arrest (CA) is one of the main causes of death in the U.S., and its impact on the healthcare system may be associated with substance-use (SU) disorders through the interplay of metabolic and cardiotoxic mechanisms. Despite the extensive research on drug overdose-related mortality, the impact of drug use on mortality from CA has not been thoroughly examined.

**Methods:**

We utilized the CDC WONDER Multiple Cause of Death database (1999–2023) to extract mortality data related to CA and SU-related causes of death among individuals aged ≥25 years. Age-adjusted mortality rates (AAMRs) per 100 000 were computed. Temporal trends were assessed using Joinpoint to calculate annual percent changes (APCs) along with 95% confidence intervals. Rates were stratified by sex, race, age groups, region, and place of death.

**Results:**

A total of 163 384 deaths were identified from 1999 to 2023. The AAMR increased from 2.22 to 4.32, surging in 2021 after a period of sharp rise (APC 13.52%, 2018–2021), before dropping thereafter (APC −7.18%, 2021–2023). Compared with women, men consistently had higher mortality rates (mean AAMR men: 4.45 vs women: 1.52). The non-Hispanic (NH) American Indian/Alaskan Natives reported the highest AAMR (9.35), whereas NH Asian/Pacific Islanders had the lowest (0.93), depicting the racial disparities. Geographic variations were notable, with the West reporting the highest burden (5.13). Most fatalities (63.3%) occurred in healthcare facilities, followed by the decedents’ homes (25.78%), with lower proportions observed in other locations.

**Conclusion:**

These findings describe a growing burden of deaths with marked sex, racial/ethnic, and geographic differences that may inform targeted prevention and equitable care delivery.

## Graphical abstract

Graphical abstract available at: https://links.lww.com/MS9/B462

## Introduction

Cardiac arrest (CA) remains one of the leading causes of mortality in the United States, as the survival outcome relies primarily on how fast recognition and intervention take place^[^1^]^. Beyond traditional risk factors like high blood pressure, diabetes, and coronary artery disease, the growing issue of substance use (SU) has emerged as a key, yet often overlooked, factor in CA deaths^[^2,3^]^. SU affects approximately 10% of the U.S. population and leads to a wide range of negative physical, mental, and social effects^[^4^]^. Substances including opioids, stimulants (e.g., cocaine and methamphetamine), alcohol, and multi-drug combinations can induce profound cardiovascular effects, such as arrhythmias and myocardial ischemia that precipitate sudden CA^[^5–7^]^.

HIGHLIGHTS

- Cardiac arrest (CA) is a leading cause of death in the U.S., and substance use (SU) significantly amplifies its burden through cardiotoxic and metabolic effects.
- This study analyzed national trends in SU-related CA mortality from 1999 to 2023 using CDC WONDER data.
- Mortality rates more than doubled (AAMR 2.22–4.32 per 100 000), peaking during 2018–2021 before a decline in 2023.
- Males, middle-aged adults (45–64 years), and non-Hispanic American Indian/Alaska Native populations had the highest mortality rates, with the Western region most affected.
- Results underscore the urgency of targeted prevention, harm-reduction strategies, and integrating SU care within cardiovascular prevention programs.

The recent few decades within the United States have witnessed multiple overlapping public health crises, including the opioid epidemic, increasing use of stimulants, and alcohol related morbidity^[^8,9^]^. These trends have coincided with significant changes in the patterns of CA, raising important questions about how much SU contributes to fatal cases. Most past research has focused on individual substances or specific groups, but wide-ranging national assessments are still lacking.

The CDC WONDER database allows us to look at long-term trends in mortality based on underlying and contributing causes of death across the U.S. population. This study explores mortality trends related to SU and CA in the United States from 1999 to 2023. By analyzing long-term patterns, demographic disparities, and the role of specific substances, our aim is to clarify the evolving role of SU in CA deaths and to help create prevention, intervention, and policy.

Although prior studies have examined SU-related cardiovascular mortality or overdose-associated CA, most have focused on specific substances, narrower time windows, or single demographic strata. In contrast, this study provides a 25-year national analysis of deaths in which SU and CA were co-listed on death certificates, with stratification by sex, race/ethnicity, age, urbanization, region, and place of death.

## Materials and methodology

### Study setting and population

Deaths occurring in the US related to both SU and CA were sourced from the CDC WONDER Multiple Cause-of-Death Public Use database. Nationwide death certificate records were analyzed to identify deaths in which both SU and CA were listed as either an underlying or contributing cause of death^[^10^]^. SU-related deaths were defined using ICD-10 codes representing SU disorders, toxic effects, poisoning or exposure, blood findings indicative of SU, and substance-related organ complications involving alcohol, opioids, cannabinoids, sedatives/hypnotics, cocaine, stimulants, hallucinogens, volatile solvents, and other psychoactive substances. Because death certificates may record different manifestations of substance-related harm, we used a broad prespecified ICD-10 definition to maximize sensitivity in this national descriptive analysis and to remain consistent with prior CDC WONDER-based approaches. The full code list is provided in Supplemental Digital Content Table S1, available at: https://links.lww.com/MS9/B408^[^9,11^]^. CA-related deaths were identified using ICD-10 code I46. Adults aged ≥25 years were included to better capture the adult population at higher risk for SU-related cardiovascular mortality while minimizing potential instability in mortality estimates among younger age groups (e.g., 18–24 years) with relatively low event counts. This study did not require approval from the Institutional Review Board because it was based on data from a de-identified database provided by the government for public use. The strengthening the reporting of observational studies in epidemiology guidelines were followed in this observational study^[^12^]^.

### Data abstraction

Data on deaths due to coexisting SU and CA, including population size and location, were gathered between January 1999 and December 2023. Demographic information, including sex and race/ethnicity, as well as state and regional data, were collected from 1999 to 2023. Race/ethnicity categories included non-Hispanic (NH) White, NH African American, Hispanic or Latino, NH American Indian or Alaska Native, and NH Asian or Pacific Islander. These classifications align with those previously used in analyses from the CDC WONDER database and are based on data reported on death certificates in compliance with the US Office of Budget and Management Guidelines^[^10^]^. The National Center for Health Statistics Urban-Rural Classification Scheme was used to classify the population into two categories based on the 2013 U.S. Census: Metropolitan (large central metro, large fringe metro, medium metro, and small metro), and non-metropolitan (micropolitan and noncore regions)^[^13^]^. U.S. Census Bureau definitions divided the regions into Northeast, Midwest, South, and West categories.

### Data synthesis

The age-adjusted mortality rates (AAMRs) and crude mortality rates (CMRs) per 100 000 population were calculated with 95% confidence intervals (CIs) to examine national mortality trends. AAMRs were determined by standardizing SU- and CA-related deaths to the 2000 U.S. standard population^[^14^]^. Age-group stratified analyses were reported as CMRs rather than age-adjusted rates because age adjustment is not meaningful within mutually exclusive age strata. To examine the long-term mortality trends, we employed the Joinpoint Regression Program (Joinpoint V 5.4.0.0, National Cancer Institute)^[^15^]^. Joinpoint regression identifies points at which statistically significant changes in mortality trends occur over time. Log-linear models were fitted to estimate annual percent change (APC) and average annual percent change with corresponding 95% CIs. A maximum of four join points were allowed, and model selection was performed using the Monte Carlo permutation method with default settings provided by the software. Considering the two-tailed *t*-test results, APCs were either increasing or decreasing if the slope representing the change in mortality deviated significantly from zero. The significance level was set at *P* < 0.05.

## Results

Between 1999 and 2023, there were a total of 163 384 deaths from CA among adults aged ≥25 years in the United States, with SU as a contributing factor (Table 1).

**Table 1:** Absolute number of substance use & cardiac arrest-related deaths per 100 000 among individuals aged 25–85 + years stratified by sex and race in the United States, 1999–2023.

Year	Overall	Women	Men	NH White	NH Black or African American	NH Asian or Pacific Islander	NH American Indian or Alaska Native	Hispanic or Latino	Population
1999	3937	939	2998	2467	746	46	66	578	180 408 769
2000	4094	989	3105	2670	721	53	46	577	181 984 640
2001	3856	998	2858	2453	683	57	53	591	184 305 128
2002	4045	986	3059	2677	633	67	59	583	186 208 028
2003	4038	1001	3037	2639	658	49	70	601	188 090 429
2004	4380	1066	3314	2862	725	71	62	641	190 205 384
2005	4494	1100	3394	2890	770	65	81	675	192 551 384
2006	4700	1224	3476	2988	782	88	91	732	195 019 359
2007	4812	1216	3596	3151	762	83	84	720	197 403 777
2008	4805	1219	3586	3200	698	78	99	721	199 795 090
2009	5016	1364	3652	3427	668	83	96	731	202 107 016
2010	5215	1447	3768	3614	649	58	114	758	203 891 983
2011	5460	1471	3989	3725	704	91	113	811	206 592 936
2012	5613	1473	4140	3867	670	81	131	838	208 826 037
2013	6163	1674	4489	4262	787	83	157	842	211 085 314
2014	6444	1756	4688	4359	807	101	170	972	213 809 280
2015	7103	1919	5184	4840	870	108	179	1057	216 553 817
2016	7626	2092	5534	5247	907	125	195	1104	218 641 417
2017	8287	2241	6046	5706	1042	123	202	1172	221 447 331
2018	8382	2250	6132	5765	1126	125	208	1123	223 311 190
2019	8794	2451	6343	5933	1273	137	184	1228	224 981 167
2020	10 906	2956	7950	7210	1541	201	308	1600	226 635 013
2021	12 539	3321	9218	8138	1734	195	365	1920	228 238 412
2022	11 782	3253	8529	7566	1668	205	332	1869	229 508 599
2023	10 893	3045	7848	7048	1548	205	236	1702	231 529 762
Total	163 384	43 451	119 933	108 704	23 172	2578	3701	24 146	5 163 131 262

### Annual trends for CA and SU-related AAMR

The AAMR for CA- and SU-related deaths in adults aged ≥25 years was 2.22 in 1999 and 4.32 in 2023. The yearly analysis of the data from 1999 to 2023 showed an overall upward trend. This trend was initially slow, with an APC of 1.08% (95% CI: 0.61–1.56) from 1999 to 2011. The increase then accelerated significantly, with the APC rising to 5.21% (95% CI: 4.01–6.42) between 2011 and 2018, and peaking at a rapid 13.52% (95% CI: 7.38–20.02) from 2018 to 2021. However, the most recent data from 2021 to 2023 show a sharp decline in the rate, with an APC of −7.18% (95% CI: −11.61 to −2.52). (Tables 1 and 2, Fig. 1, and Supplemental Digital Content Table S2, available at: https://links.lww.com/MS9/B408).

**Table 2:** APC (Annual Percent Change) of substance use & cardiac arrest-related AAMR per 100 000 in the United States from 1999 to 2023.

Year interval	APC (95% CI)
Overall	
1999–2011	1.08* (0.6–1.5)
2011–2018	5.2* (4.0–6.4)
2018–2021	13.5* (7.3–20.0)
2021–2023	−7.1* (−11.6 to −2.5)
Men	
1999–2010	0.1* (−0.4 to 0.8)
2010–2018	4.5* (3.4–5.7)
2018–2021	13.5* (6.0–21.4)
2021–2023	−7.7* (−13.1 to −2.0)
Women	
1999–2008	1.8* (0.6–3.0)
2008–2018	4.8* (3.7–5.9)
2018–2021	13.4* (3.2–24.7)
2021–2023	−5.1 (−13.4 to 4.1)
NH White	
1999–2009	2.1* (1.3–3.0)
2009–2018	5.6 * (4.6–6.6)
2018–2021	12.2* (4.4–20.6)
2021–2023	−6.6* (−13.0 to −0.2)
NH Black or African American	
1999–2012	−3.0 (−4.0 to 2.0)
2012–2018	6.0* (1.9–10.1)
2018–2021	15.9* (1.7–32.1)
2021–2023	−6.2 (−17.0 to −6.0)
NH American Indian or Alaska Native	
1999–2018	5.2 (4.1–6.3)
2018–2021	24.7* (−0.4 to 56.3)
2021–2023	−17.2 (−32.6 to 1.7)
Hispanic or Latino	
1999–2009	−2.3* (−3.1 to −1.4)
2009–2018	0.6* (−0.2 to 1.6)
2018–2021	13.7* (5.6–22.3)
2021–2023	−7.0 (−12.7 to −1.0)
NH Asian or Pacific Islander	
1999–2013	−1.9 (−4.1 to 0.2)
2013–2023	6.4* (3.9–9.0)
Nonmetropolitan areas	
1999–2018	4.0* (3.6–4.5)
2018–2020	17.6* (5.1–31.7)
Metropolitan area	
1999–2011	0.2* (−0.5 to 1.1)
2011–2020	6.2* (5.1–7.3)
25–44 Years	
1999–2012	1.56* (0.76–2.37)
2012–2018	7.08* (4.17–10.06)
2018–2021	18.20* (6.21–31.54)
2021–2023	−8.60* (−16.27 to −0.23)
45–64 Years	
1999–2010	1.92* (1.30–2.55)
2010–2018	4.88* (3.89–5.88)
2018–2021	12.00* (5.56–18.83)
2021–2023	−8.18* (−13.33 to −2.71)
65–85 + Years	
1999–2008	−2.74* (−4.19 to −1.27)
2008–2018	4.50* (3.14–5.87)
2018–2021	12.54* (1.03–25.36)
2021–2023	−2.04 (−10.94 to 7.76)
Northeast region	
1999–2007	1.0*(−1.3 to 3.4)
2007–2011	−4.6* (−14.8 to 6.8)
2011–2016	8.0* (1.3–15.2)
2016–2023	2.6* (−1.6 to 7.0)
Midwest region	
1999–2008	1.5* (0.3–2.7)
2008–2017	8.2* (7.1–9.4)
2017–2020	15.9* (8.0–24.4)
2020–2023	−21.6 (−33.7 to −7.2)
South region	
1999–2012	0.5* (−0.2 to 1.4)
2012–2020	7.0* (5.1–8.9)
2020–2023	−19.0* (−32.4 to −3.0)
West region	
1999–2018	2.1* (1.5–2.8)
2018–2023	14.0* (8.3–20.0)

*Statistically significant values, defined as *P* < 0.05.

**Figure 1.**
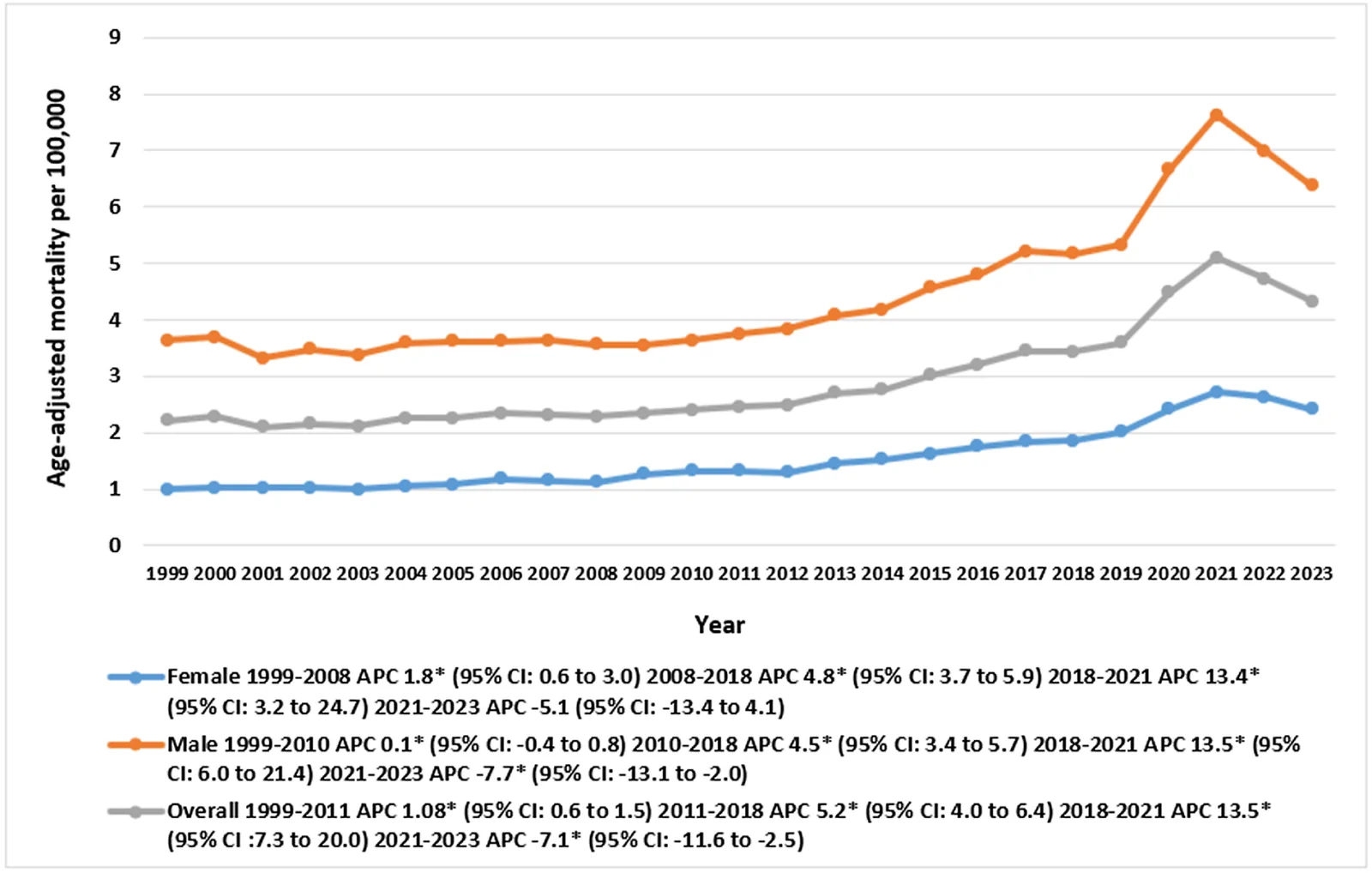
Overall and sex stratified age-adjusted mortality rates per 100 000 for SU and CA-related mortality in United States adults aged ≥25 years, 1999–2023. APC, annual percentage change; CI, confidence interval. * The annual percentage change (APC) is significantly different from zero at *α* = 0.05.

### CA and SU-related AAMR stratified by sex

The AAMR for both males and females exhibited a similar pattern over time, but at different scales. Throughout the study period, males consistently maintained a higher AAMR than females (mean overall AAMR males: 4.45; females: 1.52). The AAMR for males at the start of the period was 3.63, whereas females had an AAMR of 1 in 1999. The trend started with a period of relative stability for both males and females and reached an AAMR of 3.64 (in 2010) for males and 1.13 (in 2008) for females. This then followed a period of moderate growth until 2018 (male APC: 4.59; 95% CI: 3.49–5.70; female APC: 4.85; 95% CI: 3.77–5.93), before a sharp acceleration in rates from 2018 to 2021 (male APC: 13.51; 95% CI: 6.07–21.45; female APC: 13.45; 95% CI: 3.21–24.7). However, the most recent period from 2021 to 2023 shows a significant reversal, with both rates declining, with the male rate dropping more sharply at an APC of −7.78% (95% CI: −13.18 to −2.04) compared to the female rate at −5.10% (95% CI: −13.49 to −4.10). The AAMRs for both males and females at the end of the study period were 6.37 and 2.42, respectively (Tables 1 and 2, Fig. 1, and Supplemental Digital Content Table S2, available at: https://links.lww.com/MS9/B408).

### CA and SU-related AAMR stratified by race

When stratified by race, the AAMRs were highest among NH American Indian or Alaska Native patients, followed by Hispanic or Latino, NH Black or African American, NH White, and NH Asian or Pacific Islander populations [mean overall AAMR NH American Indian or Alaska Native: 9.35 (95% CI: 7.85–10.96); Hispanic or Latino: 3.92 (95% CI: 3.66–4.19); NH Black or African American: 3.69 (95% CI: 3.45–3.94); NH White: 2.78 (95% CI: 2.70–2.87); NH Asians or Pacific Islanders: 0.93 (95% CI: 0.75–1.15)]. From 1999 to 2018, mortality rates increased for NH African Americans and NH Whites and declined for all other groups. While all racial groups experienced an increase up to 2021, the American Indian or Alaska Native population had the most prominent rise among all the racial groups, with an exceptionally high APC of 24.76% (95% CI: −0.43 to 56.3) from 2018 to 2021 before a steep decrease. In contrast, the Asian or Pacific Islander group maintained a relatively flat and stable rate throughout the period, with much less pronounced changes compared to the other groups. (Tables 1 and 2, Fig. 2, and Supplemental Digital Content Table S3, available at: https://links.lww.com/MS9/B408)

**Figure 2.**
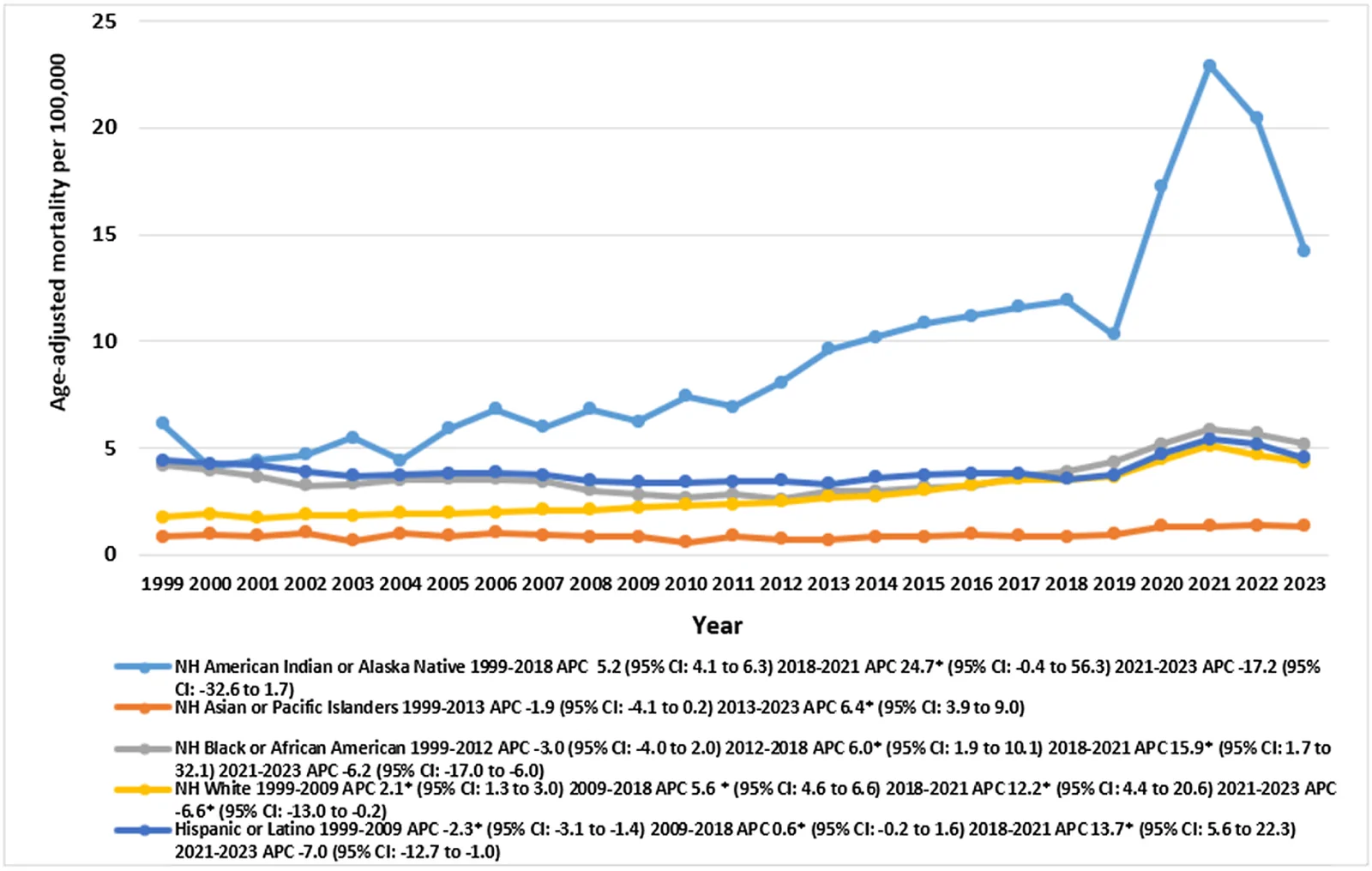
Race stratified age-adjusted mortality rates per 100 000 for SU and CA-related mortality in United States adults aged ≥25 years, 1999–2023. APC, annual percentage change; CI, confidence interval. * The annual percentage change (APC) is significantly different from zero at *α* = 0.05.

### CA and SU-related AAMR stratified by age group

CA and SU-related mortality rates varied significantly across age groups. Adults aged 25–44 years had the lowest CMR per 100 000 population (overall mean CMR 1.42; 95% CI: 1.34–1.50). Adults aged 65–85+ years had an intermediate mean CMR of 3.71 (95% CI: 3.54–3.89), whereas adults aged 45–64 years experienced the highest mortality burden, with an overall mean CMR of 4.56 (95% CI: 4.41–4.71). Because these age strata are mutually exclusive, these values are presented as CMRs rather than age-adjusted rates (Table 2, Fig. 3, and Supplemental Digital Content Table S4, available at: https://links.lww.com/MS9/B408).

**Figure 3.**
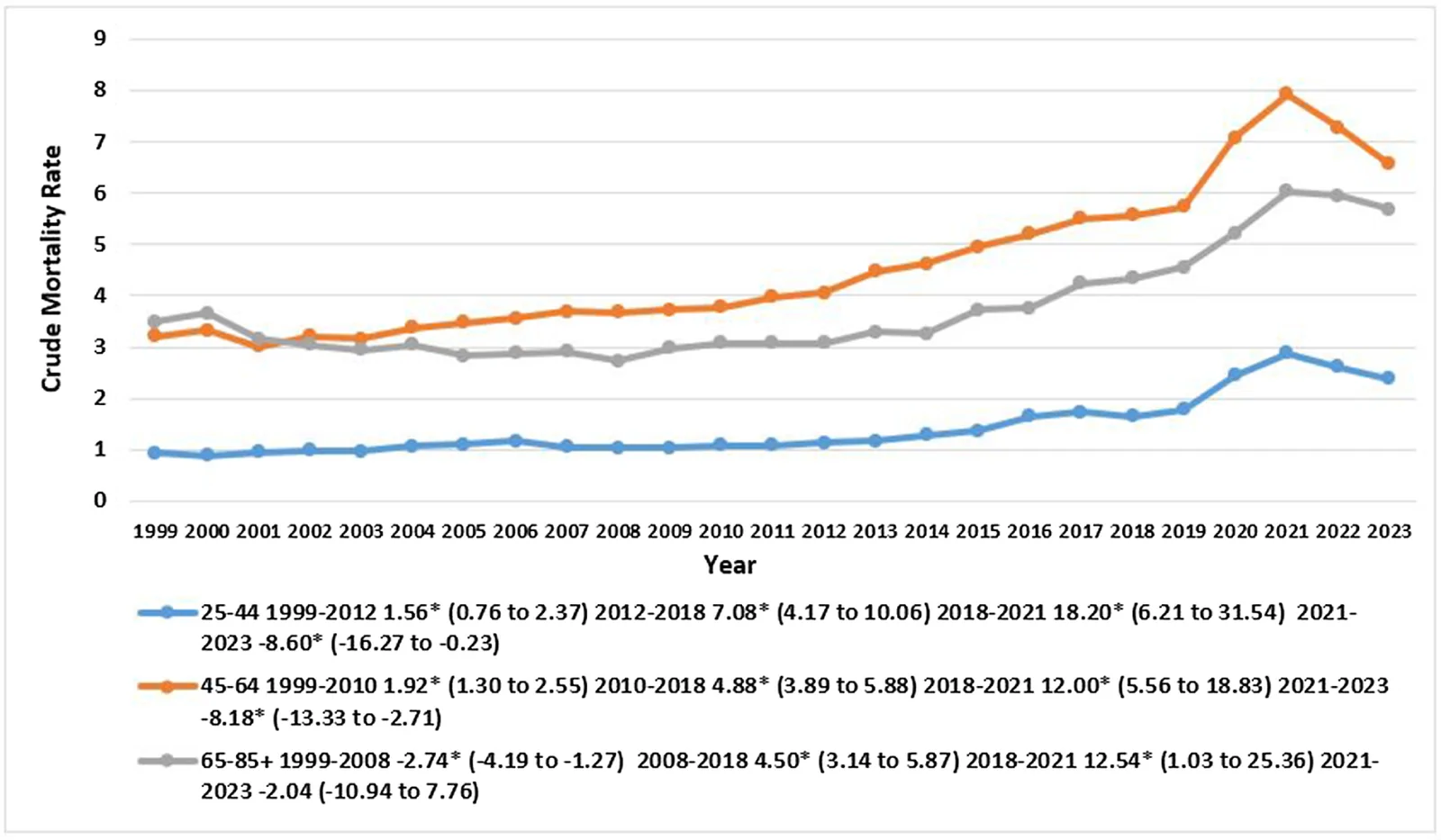
Age group stratified crude mortality rates per 100 000 for SU and CA-related mortality in United States adults aged ≥25 years, 1999–2023. APC, annual percentage change; CI, confidence interval. * The annual percentage change (APC) is significantly different from zero at *α* = 0.05.

### CA and SU-related AAMR stratified by urbanization

Data for urbanization analysis were collected from 1999 to 2020. Over this period, non-metropolitan areas consistently had a higher AAMR than metropolitan areas, with overall AAMRs of 3.16 (95% CI: 3.12–3.2) and 2.65 (95% CI: 2.63–2.66), respectively. Both areas experienced a sharp and significant surge in rates over the years. The non-metropolitan trend showed a significant increase from 1999 to 2018 (APC: 4.06; 95% CI: 3.60–4.52), before its final surge up to 2020 (APC: 17.65; 95% CI: 5.10–31.71). In contrast, the metropolitan areas experienced an initial period of stabilization from 1999 to 2011 (APC: 0.30; 95% CI: −0.54 to 1.14), followed by a significant increase from 2011 to 2020 (APC: 6.24; 95% CI: 5.18–7.32) (Table 2, Fig. 4, and Supplemental Digital Content Table S5, available at: https://links.lww.com/MS9/B408).

**Figure 4.**
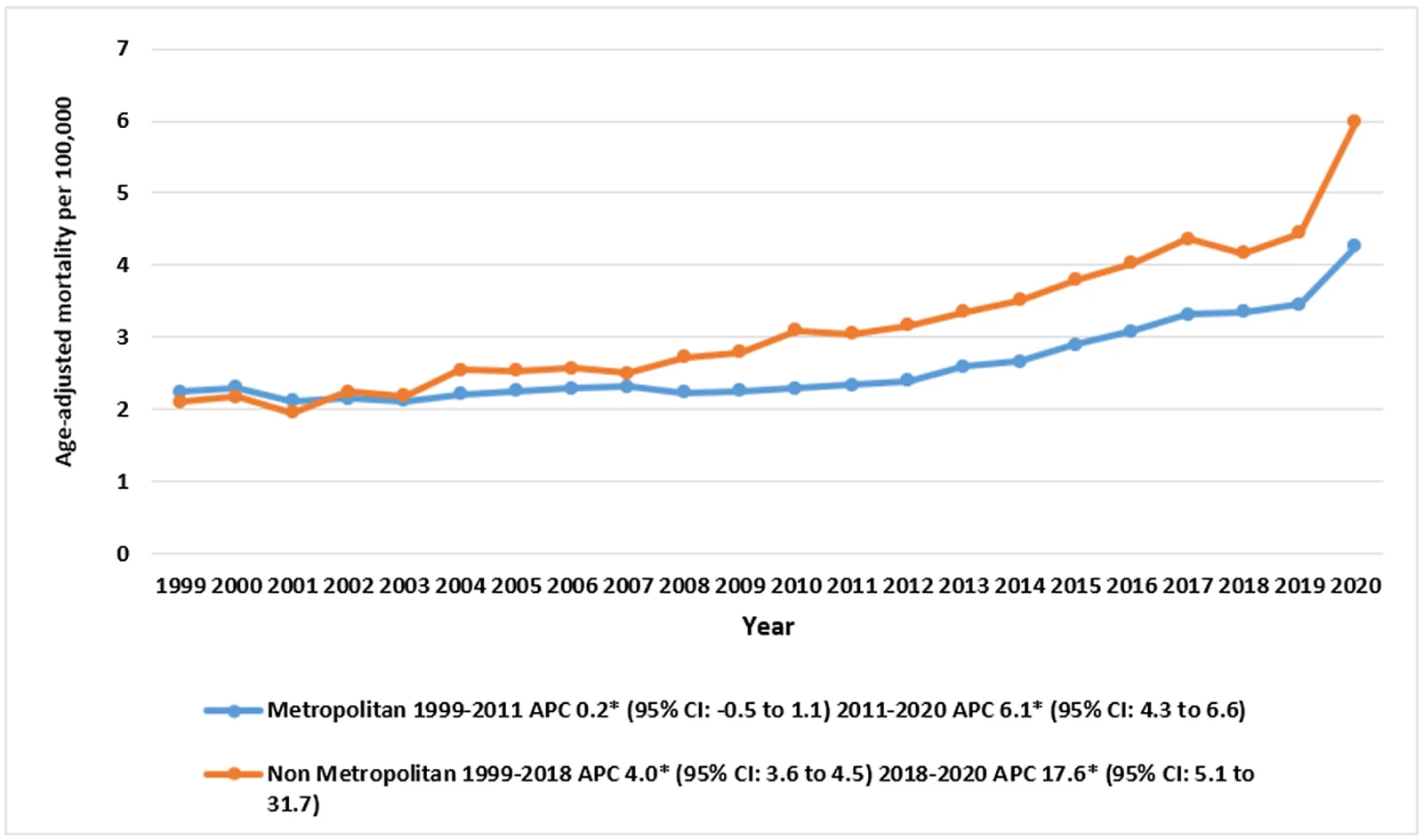
Urbanization stratified age-adjusted mortality rates per 100 000 for SU and CA-related mortality in United States adults aged ≥25 years, 1999–2023. APC, annual percentage change; CI, confidence interval. * The annual percentage change (APC) is significantly different from zero at *α* = 0.05.

### CA and SU-related AAMR stratified by census regions

Over the course of the study, the highest mortality was observed in the Western region (mean AAMR: 5.13; 95% CI: 4.89–5.36), followed by the Northeastern (mean AAMR: 2.75; 95% CI: 2.55–2.95), Southern (mean AAMR: 2.15; 95% CI: 2.03–2.27), and Midwestern regions (mean AAMR: 1.73; 95% CI: 1.56–1.90). The Western region showed a consistent upward trend from 1999 to 2018 with an APC of 2.19 (95% CI: 1.53–2.86), before increasing dramatically from 2018 to 2020 (APC: 14.03; 95% CI: 8.31–20.04). The trend for the Northeast region showed multiple shifts. It started with a gradual increase of 1.04% (95% CI: −1.31 to 3.46) between 1999 and 2007, followed by a slight decline before finally exhibiting a sharper increase in the last few years. Both the Midwestern and Southern regions showed a relative stabilization phase in the initial years of the study, with subsequent significant variations. The Midwestern region showed a significant increase between 2017 and 2020 (APC: 15.95; 95% CI: 8.02–24.46), before experiencing a final decline between 2020 and 2023 (APC: −21.60; 95% CI: −33.72 to −7.27). In contrast to that, the Southern region showed a slight incline between 2012 and 2020 (APC: 7.04; 95% CI: 5.16–8.95), which was followed by a sharp decline in the final years from 2020 to 2023 (APC: −19.06; 95% CI: −32.46 to −3.01). (Table 2, Fig. 5, and Supplemental Digital Content Table S6, available at: https://links.lww.com/MS9/B408).

**Figure 5.**
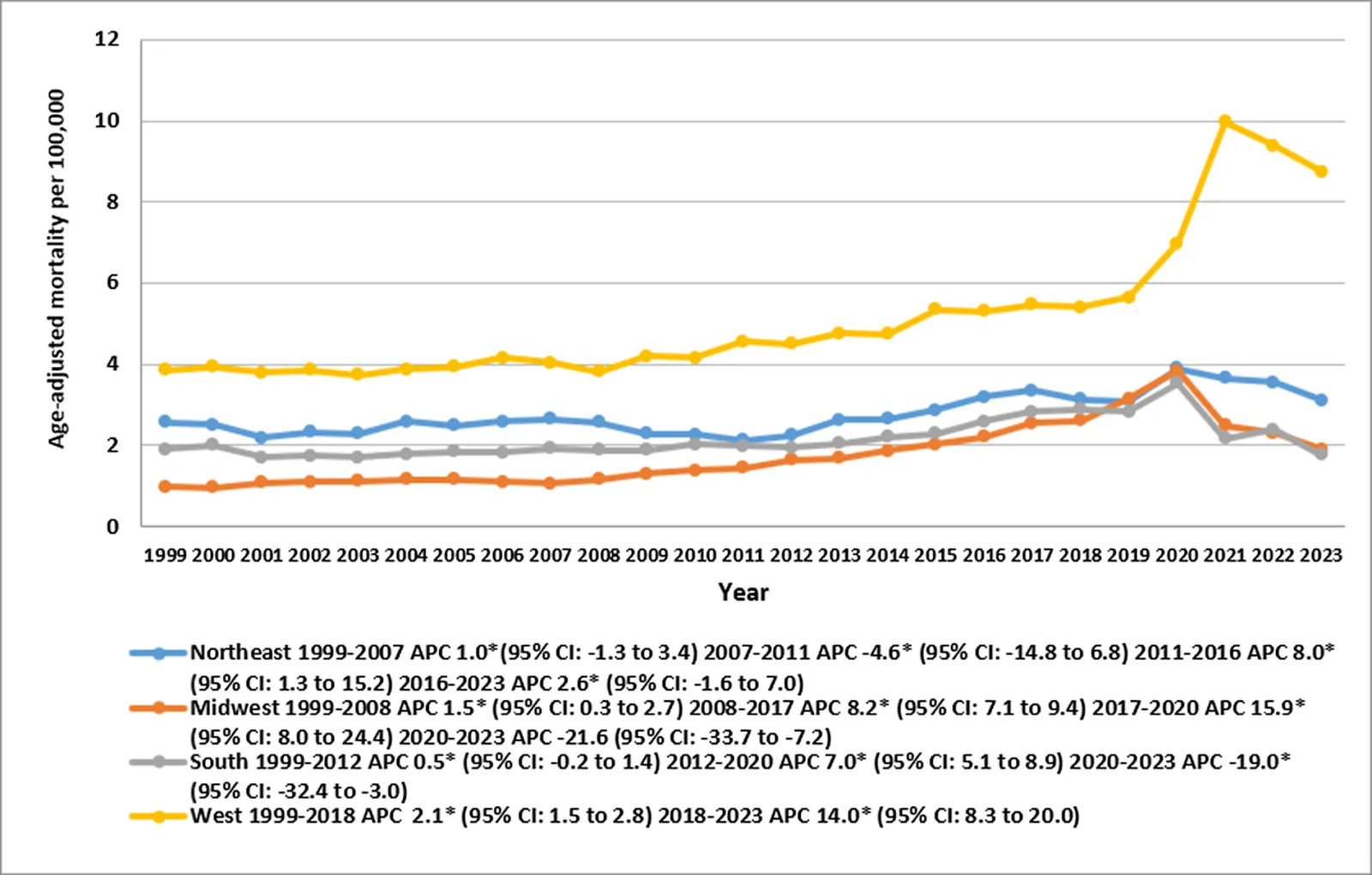
Census region stratified age-adjusted mortality rates per 100 000 for SU and CA-related mortality in United States adults aged ≥25 years, 1999–2023. APC, annual percentage change; CI, confidence interval. * The annual percentage change (APC) is significantly different from zero at *α* = 0.05.

### CA and SU-related AAMR stratified by states

Between 1999 and 2023, significant variation in AAMRs was observed across states, with the lowest rate in Maine (mean AAMR: 1.14) and the highest in Mississippi (mean AAMR: 10.56). States falling within the top 10th percentile for mortality rates included Mississippi (mean AAMR: 10.56), Alaska (mean AAMR: 8.40), California (mean AAMR: 8.06), Nevada (mean AAMR: 6.05), and South Dakota (mean AAMR: 5.96) while those in lowest 10th percentile were Maine (mean AAMR: 1.14), Virginia (mean AAMR: 1.42), Oklahoma and Texas (mean AAMR tied at 1.8), and Illinois (mean AAMR: 1.82) (Supplemental Digital Content Figure S1, available at: https://links.lww.com/MS9/B408 and Supplemental Digital Content Table S7, available at: https://links.lww.com/MS9/B408).

### CA and SU-related AAMR stratified by place of death

Information for the location of death was available for 163 006 deaths. A significant majority of these deaths took place in medical facilities (63.31%). The next most common location was the decedents’ homes, accounting for 25.78% of the total deaths. The remaining fatalities were spread across nursing homes (5.44%), hospice fatality (1.37%), and other locations (3.87%) (Supplemental Digital Content Figure S2, available at: https://links.lww.com/MS9/B408 and Supplemental Digital Content Table S8, available at: https://links.lww.com/MS9/B408).

### Sensitivity analysis

We performed a sensitivity analysis to assess whether the observed pattern in the CA-SU intersection was consistent with the broader SU mortality trend. Over the 25-year study period, the AAMR for the total SU cohort increased from 30.02 to 85.4 per 100 000, while the CA-SU intersection increased from 2.22 to 4.32 per 100 000. Both datasets showed broadly similar temporal inflection points, including a peak in 2021 followed by a modest decline thereafter. This concordance supports the internal consistency of the observed pattern, although it does not establish causality or exclude the influence of coding and compositional differences. (Supplemental Digital Content Figure S3, available at: https://links.lww.com/MS9/B408 and Supplemental Digital Content Table S9, available at: https://links.lww.com/MS9/B408).

## Discussion

This study descriptively analyzes mortality trends among U.S. individuals aged 25 years and above in whom SU and CA were both listed among the causes of death. Certain key trends are particularly noteworthy. A rise in mortality across every stratum was observed during 2018–2021. During this period, the Western region reflected the highest mean AAMR, while individuals aged 45–64 years demonstrated higher mortality rates compared to other age groups. All racial groups exhibited an increase in AAMR up to 2021, with NH Alaskan Natives or American Indians experiencing the most prominent rise. Mortality rates also increased significantly in both urban and rural counties. Notably, there was a decline in AAMRs among all strata from 2021 to 2023. Overall, the findings describe distinct geographic, demographic, and racial disparities in SU- and CA-related mortalities, with a clear rise until 2021, followed by a decline thereafter.

Our analysis shows that the trends before 2019 were generally less stark and concordant with contemporary literature, but the mortality rates increased most sharply during 2018–2021 (APC: 13.52), and declined thereafter^[^16^]^. This temporal pattern coincided with the COVID-19 period and may reflect contemporaneous disruptions in healthcare access, social conditions, and the drug supply; however, the present design cannot determine whether any of these factors directly drove the observed trend. Similar trends have been reported in earlier studies, with one such nationwide study evaluating methadone use pre- and post-2019. Overdoses increased from 1999 through the early 2000s, peaked in 2007, and declined until 2019. Overdoses in 2020 were 48.1% higher than in 2019, and previous studies have reported cardiac abnormalities in association with methadone use^[^17,18^]^. A similar trend was seen with other scheduled narcotic overdose rates, with published year-on-year rates ranging from 33.7 to 50% when comparing the 12-month period before and after March 2020^[^18^]^. Honing in on CA data, an analysis of the US National Emergency Medical Services Information System database showed that overdose-related CAs reached their highest count for a single month in May 2020^[^19^]^.

The observed increase during 2018–2021 coincided with the COVID-19 period, but this ecological design cannot determine whether pandemic-related disruptions directly drove the trend. Changes in healthcare access, social conditions, and the drug supply are plausible contextual factors, but they are not directly measured in CDC WONDER. Prior literature has described associations between social isolation, reduced healthcare access, and SU risk; however, these factors cannot be directly assessed in the present database. Reduced healthcare access during the pandemic has been documented, with total patient encounters decreasing from 2.86 million in March 2020 to a low of 2.35 million in April 2020, while the incidence of CAs was also reported to be increasing^[^19^]^. Our findings are broadly consistent with prior population-level studies that have reported a decline in mortality rates in the post-2021 period [APC: −7.18% (95% CI: −11.61 to −2.52)]. The overall trend is mirrored when stratified by sex, for largely the same reasons discussed above. Trends stratified by age point to a higher CMR among older individuals. The higher crude mortality observed among older adults may be consistent with differences in comorbidity burden and frailty reported in prior literature; however, these factors could not be directly assessed in the present database. [CMR: 4.56 (95% CI: 4.41–4.71)]^[^20–22^]^.

Regional differences may reflect variation in SU patterns, access to emergency and preventive care, and harm-reduction infrastructure. However, because CDC WONDER does not provide individual-level socioeconomic or clinical data, these explanations remain speculative and should be interpreted cautiously. Western mortality rates (mean AAMR: 5.13; 95% CI: 4.89–5.36) in our analysis align with previous state- and county-level studies. As a microcosm of the larger trend, San Francisco serves as a case study. Paramedical databases show that while drug-related overdoses comprised 1% of CAs in 2015, the rate had grown exponentially to 17.6% in 2023. Furthermore, fentanyl availability has been identified as an important contextual factor in prior studies, with changes in the drug supply reported during the COVID-19 period^[^23,24^]^. However, these factors were not directly measured in the present analysis^[^23,24^]^. The same synthetic opioid has been associated with increased overdose-related harm in Southern states, with prior studies suggesting that street drug use is the primary driver rather than prescription abuse^[^25,26^]^. The Northeast, particularly the Appalachian region, has also been disproportionately affected by the opioid crisis. Persistent Medicaid non-expansion, higher-than-average poverty rates, and healthcare access challenges have been described in this region and may provide relevant contextual considerations for interpreting the observed mortality patterns^[^27,28^]^. More generally, previous literature has discussed the rising prevalence and distribution of opioids in the years leading up to the pandemic. However, the subsequent decline coincided temporally with increased implementation of harm-reduction measures, such as safe-use sites and fentanyl test strips. Because these interventions were not directly assessed in the present analysis, their contribution to the observed decline cannot be determined^[^29,30^]^.

Marked ethnic disparities were identified across groups, consistent with previously reported structural and healthcare inequities. The trend of an increase in mortality in NH Whites and African Americans from 1999 to 2018, in contrast to decreases across all other races in the same time period, may be associated with patterns described during the “second” and “third waves” of the opioid crisis in urban populations as discussed in prior studies. However, the fact that overall NH Whites comprise one of the lowest AAMRs is of note, indicating that overdose-related mortality is not a “White problem”^[^31^]^. Indeed, literature has also extensively highlighted the barriers to healthcare access in minority communities, with studies impressing upon the fact that the Indian Health Service has been historically underfunded, with the per capita expenditure on patients being $4078 as compared to the national average of $9726, and beset by challenges including intra-group historical trauma and increased risk of depression and suicide, with prior literature describing historical trauma, barriers to healthcare, and disparities in mental health outcomes among Indigenous populations^[^32–34^]^. Importantly, the pronounced increase among NH American Indian or Alaska Native decedents may reflect a combination of historical inequities, access barriers, and reporting differences, but the present data cannot quantify the contribution of any single factor. Concurrently, a surge in synthetic opioid and polysubstance availability, coupled with reduced emergency response capacity in rural tribal regions, may have contributed to increased mortality during this period. The subsequent decline coincided with broader restoration of healthcare access, implementation of targeted tribal overdose-prevention programs, and adaptation of telehealth and community-based interventions in the post-pandemic period^[^35–37^]^. Prior studies have reported widespread social and healthcare disruptions during the COVID-19 pandemic, including increased social isolation and reduced access to support groups, healthcare services, and harm-reduction facilities. These factors may have contributed to the increases in mortality observed across racial groups during the 2018–2021 period, followed by declining trends after 2021^[^38^]^. Differences in access, population composition, coding patterns, and unmeasured social factors may contribute to the observed stability^[^39^]^.

### Policy implications

These findings support targeted, equity-focused prevention efforts, improved access to treatment and overdose-reversal services, and continued population-level surveillance. However, because the present study is ecological and based on death-certificate data, it cannot determine which specific interventions caused changes in mortality. The results identify populations and regions that may merit prioritization in future public health planning.

### Study limitations

The study possesses several limitations, primarily due to its retrospective design. Because CDC WONDER is based on death-certificate data, misclassification of both CA and SU is possible, and SU may be underreported on death certificates. Coding practices may also change over time, which can affect temporal comparisons. The study cannot establish temporality or causality at the individual level. The reliance on ICD-10 codes may introduce misclassification, and the broad SU definition used in this study was intended to maximize sensitivity but may limit clinical specificity. CDC WONDER also lacks individual-level information on socioeconomic status, comorbidities, treatment exposure, and toxicological findings, precluding assessment of specific clinical circumstances or determination of whether SU directly precipitated CA. Furthermore, we used the 2000 U.S. standard population for calculating the AAMRs. This may reduce comparability over a 25-year period, especially due to the change in demographics that occurred during this time. Given the use of 2021 population estimates, the provisional AAMRs likely underestimate SU and CA deaths and trends, and excess deaths may be even higher than reported here. Furthermore, the data extracted for urban stratification were limited to 2020. However, as compared to existing literature, our study utilized a more inclusive and broader category of codes to include a large population with a history of SU^[^40^]^; the accuracy of these codes could not be independently verified. In addition, the database lacks individual-level information on socioeconomic status, comorbidities, treatment exposure, and other confounders, so multivariable adjustment was not possible. Accordingly, the findings should be interpreted as descriptive ecological associations rather than causal effects. Additionally, in our analysis, smoking was not assessed as a contributor to SU-related deaths because of the primary focus on other substances, and we could not perform multivariable analyses. Spanning the years 1999–2023, the findings provide a long-term view of how mortality statistics related to SU and CA have changed over more than two decades.

## Conclusion

Overall, deaths linked to SU and CA have more than doubled over the past two decades, revealing significant demographic, geographic, and temporal disparities. The crisis disproportionately affected middle-aged adults, males, and NH American Indian or Alaska Native populations, reflecting deeper issues with health equity and access to preventive care. The persistent burden in rural and Western areas highlights uneven socioeconomic conditions and health infrastructure, demanding sustained attention. Integrating the management of SU into cardiovascular prevention programs, alongside targeted, evidence-based policy measures such as harm-reduction initiatives and equitable healthcare access, is crucial to reducing the burden of SU- and CA-related deaths in the United States.

## Data Availability

The datasets generated and/or analyzed during the current study are available from the corresponding author on reasonable request.

## Study participants

The research is based on de-identified human death certificate data from the CDC WONDER database. https://wonder.cdc.gov/mcd.html.
